# The effect of combined treatment with morphine sulphate and low-dose ketamine in a prehospital setting

**DOI:** 10.1186/1757-7241-17-61

**Published:** 2009-11-27

**Authors:** Patric Johansson, Poul Kongstad, Anders Johansson

**Affiliations:** 1 Department of Falck Ambulance Ltd, Linnegatan 2, 281 25, Hässleholm, Sweden; 2 Department of Prehospital Care and Disaster Medicine in Region of Skane, Box 1, 221 01 Lund, Sweden; 3 Department of Health Sciences, Faculty of Medicine, Lund University, PO Box 157, SE-221 00 Lund, Sweden

## Abstract

**Background:**

Pain is a common condition among prehospital patients. The present study is designed to determine whether adding low-dose ketamine as additional analgesia improves the pain/nausea scores and hemodynamic parameters compared to morphine sulphate alone among patients with bone fractures.

**Methods:**

Prospective, prehospital clinical cohort study. Twenty-seven patients were included with acute pain. Eleven patients received morphine sulphate 0.2 mg/kg (M-group) and 16 patients received morphine sulphate 0.1 mg/kg combined with 0.2 mg/kg ketamine (MK-group). Scores for pain, nausea, sedation (AVPU) and the haemodynamic parameters (systolic blood pressures (BP), heart rate (HR) and peripheral oxygen saturation (SpO2) were recorded at rescue scene before the start of analgesia and subsequently to admission at hospital.

**Results:**

Mean treatment time 46 ± 17 minutes in the M-group and 56 ± 11 minutes in the MK-group, respectively (ns). Mean doses of morphine sulphate in the M-group were 13.5 ± 3.2 mg versus 7.0 ± 1.5 mg in the MK-group. The mean additional doses of ketamine in the MK-group were 27.9 ± 11.4 mg. There were significantly differences between the M- and the MK-group according to NRS scores for pain (5.4 ± 1.9 versus 3.1 ± 1.4) and BP (134 ± 21 mmHg versus 167 ± 32 mmHg) at admission at hospital, respectively (*P *< 0.05). All patients were Alert or respond to Voice and the results were similar between the groups. One patient versus 4 patients reported nausea in the M- and MK-group, respectively, and 3 patients vomited in the Mk-group (ns).

**Conclusion:**

We conclude that morphine sulphate with addition of small doses of ketamine provide adequate pain relief in patients with bone fractures, with an increase in systolic blood pressure, but without significant side effects.

## Background

In Sweden since 2005, there is a requirement of at least one licensed nurse per emergency ambulance. It appears in the skill description of nurses in Sweden, that specialised ambulance nurses must have extended knowledge in medicine and nursing care [1]. A specialist nurse in prehospital care must be able to perform and be responsible for examination and treatment of acute pain in the special prehospital area [2].

Pain is a common condition among prehospital patients. A literature review 2008 by Thomas and Shewakramani confirmed that there is evidence supporting the safety of prehospital analgesia, however they also conclude that different providers should assess available information to further improve pain relief [3]. Analgesia's importance is magnified by the frequency with which different emergency providers interact with injured patients. Moderate or severe pain is present in 80% of patients with extremity fractures [4].

Drug options generally available in the prehospital area include morphine, fentanyl, tramadol, ketorolac and ketamine [3]. For simple analgesia morphine sulphate is usually effective, however often preceded by an antiemetic agent. Another option for patients with various injuries and those requiring manoeuvring and splinting is ketamine. Ketamine offers a safe and effective analgesia since this agent avoids the potential decrease in blood pressure and respiratory depression that is associated with opioid analgesia [5-7].

The present study is designed to determine whether adding low-dose ketamine as additional analgesia improves the pain/nausea scores and hemodynamic parameters compared to morphine sulphate alone in a prehospital setting among patients with bone fractures.

## Methods

Following ethics committee approval this study was carried out in (Region of Skane) southern Sweden, for the period of spring and autumn 2008. Study design was a prospective, clinical cohort study with a random inclusion. Patients with bone fractures were randomly assigned to one of two treatments (11 patients in each group), to receive morphine sulphate intravenously in M-group (*n *= 11), and the other group MK (*n *= 11) received morphine sulphate plus ketamine. To possibly detect other (not known-) effects in the MK-group an extra 5 patients were included in this group (total *n *= 16). Exclusion criteria include the inability to use the rating scale, long-term use of opioids, history of chronic pain, history of/or acute myocardial infarction and unconsciousness. Every five minutes monitoring includes pulse oximetry, automated blood pressure, heart rate (HR), breathing frequencies (AR) and lead II electrocardiogram. The breathing frequencies were measured during 60 seconds every five minutes.

At the same time-interval (every five minutes) Numeric Rating Scale (NRS) was used for pain and nausea assessment (NRS, 1 = no pain/nausea, 10 = worst pain/nausea). In all patients, when the NRS scores for pain were four (≥ 4) or greater, a standardized (0.1 mg/kg) loading dose of morphine sulphate was given. Subsequently (every five minutes), if patients still report NRS scores four or greater, the patients in the M-group received a supplementary dose of morphine sulphate to a total dose of 0.2 mg/kg. In the MK-group the patients received 0.2 mg/kg ketamine doses instead of the supplementary dose of morphine sulphate in the M-group, to maintain NRS scores below four.

Scores for pain, nausea, sedation (AVPU) and the haemodynamic parameters (systolic blood pressures (BP), heart rate (HR) and peripheral oxygen saturation (SpO2) were recorded at rescue scene before the start of analgesia and subsequently to admission at hospital.

During the evaluations of the pain/nausea scores, the nurses documented if the treated patients could respond adequately. This was done using a 4- point sedation scale (AVPU = 1-Alert, 2-respond to Voice, 3-respond to Pain, 4-Unresponsive) [8]. Treatment time, total and bolus doses of morphine and/or ketamine, side-effects (such as sedation AVPU > 2, or hallucinations), frequencies of nausea and vomiting associated with present procedure were recorded.

### Statistics

The results are presented as mean, standard deviations (SD), median and quartiles. Demographic data were analysed using parametric t-test. Pain and nausea scores were analysed using non-parametric test (Mann-Witney) and sedation, nausea and vomiting scores were analysed using Chi-Square tests. An initial power analysis showed that with a clinical relevant difference in NRS-scores of 1 for pain, with a SD of 0.75, reaches a power-value of 0.8 with 9 patients included in each group against a p-value of 5%. *P *< 0.05 is considered statistically significant [9]. Data analysis and statistical calculations were performed using SPSS version 14.5 (SPSS Inc., Chicago, IL).

## Results

The data collection included 27 patients, 11 patients in the M-group versus 16 patients in the MK-group. Demographic data, type of fractures and treatment times are shown in Table 1. Besides nausea and vomiting, there were no adverse drug effects during the treatment with morphine sulphate and/or ketamine.

**Table 1:** Demographics

	M-group	MK-group
	n = 11	n = 16
Sex		
Male	6	7
Female	5	9

Age (year)	70 ± 16	74 ± 14

Weight (kg)	72.9 ± 13.6	70.1 ± 10.4

Treatment times (minutes)	46 ± 17	56 ± 11

Type of fractures (n)		
Hip	5	7
Femur	1	3
Lower limb	2	2
Shoulder	1	2
Upper arm	2	1
Forearm	0	1

Nausea	1	4

Vomiting	0	3

Sedation (AVPU > 2)	0	0

Hallucinations	0	0

Demographic data, treatment times, type of fractures, frequencies of Nausea, Vomiting, Sedation and Hallucinations. Values are presented as frequencies and mean ± SD and demonstrates no differences within- and between the groups (* = p < 0.05).

Mean doses of morphine sulphate in the M-group were 13.5 ± 3.2 mg versus 7.0 ± 1.5 mg in the MK-group, which is in accordance with the average weights of the patients. The mean additional doses of ketamine in the MK-group were 27.9 ± 11.4 mg. The NRS scoring for pain in the prehospital period was similar in the groups at arrival to the scene (Table 2). There were significantly differences between the M- and the MK-group according to BP and NRS at admission to hospital, respectively (*P *< 0.05) (Table 2 and 3).

**Table 2:** NRS scores for pain at rescue scene and at admission to hospital.

	NRS at scene		NRS at admission hospital	
	**M-group**	**MK-group**	**M-group**	**MK-group**

Mean	8.5	7.5	5.4*	3.1*

Median	9.0	7.5	5.0*	3.0*

SD.	1.6	1.8	1.9	1.4

Percentiles 25	7.0	6.0	4.0	2.0

50	9.0	7.5	5.0	3.0

75	10.0	9.5	7.0	3.8

Statistical differences were found between the M-group and the MK-group (* = p < 0.05).

**Table 3:** Frequencies of the measured variables

	M-group	MK-group
	n = 11	n = 16
BP rescue scene (mmHg)	143 ± 17	141 ± 33

BP admission to hospital (mmHg)	134 ± 21	167 ± 32*

HR rescue scene (beat/min)	74 ± 11	82 ± 17

HR admission to hospital (beat/min)	72 ± 9	78 ± 13

SpO_2 _rescue scene (%)	96 ± 2.6	94 ± 5.3

SpO_2 _admission to hospital (%)	97 ± 2.1	98 ± 1.8

AR rescue scene (breath/min)	17 ± 3	18 ± 6

AR admission to hospital (breath/min)	16 ± 3	18 ± 5

Haemodynamic parameters (systolic blood pressures (BP), heart rate (HR), peripheral oxygen saturation (SpO_2_) and breath per minute (AR) at rescue scene and at admission to hospital. Values are presented as mean ± SD (* = p < 0.05).

All patients were Alert or respond to Voice using the AVPU-scale (Table 1). The number of patients suffering from adverse events is shown in Table 1, describing 1 patient versus 4 patients reported nausea in the M- and MK-group (ns), respectively, and 3 patients vomited in the MK-group (ns).

## Discussion

The purpose of this study was to evaluate whether adding low-dose ketamine to a standard morphine sulphate dose improves the pain/nausea scores and hemodynamic parameters compared to morphine sulphate alone in a prehospital setting in patients with bone fractures. This study shows adequate analgesia from small doses of additional ketamine, with stable vital parameters, however with a tendency of increased frequency of nausea and vomiting. The used combination is in accordance with other studies that shows similar clinically relevant opioid sparing effects [10,11].

Demographic data show an equal distribution of men and women in both groups and descriptive data showing comparable readings on most variables. The average dose of morphine sulphate in both the M- and MK-group are in line with the designed doses, ie. 0.2 mg/kg in the M-group versus 0.1 mg/kg of the MK-group. This is normal doses of morphine sulphate available on the general delegation in ambulance care in southern Sweden [2]. The total dose of ketamine per kg (≈30 mg) corresponding to around 0.4 mg/kg. Since the design of the additional doses of ketamine was 0.2 mg/kg this is in relation to 2-3 doses of ketamine in the nursing care situation of about 50 minutes. We believe that this reflects reality quite well.

Heart rate, SpO_2 _and respiratory rate were stable vital parameters and similar between the groups. In this study there are no significant differences but notably is that most parameters except heart rate and NRS scores, are increased in the MK-group. NRS values for pain during admission to hospital was significantly lower in the MK-group, and this value (3.1 ± 1.4) is satisfying since the Swedish Association of Anaesthesia and Intensive care (SFAI) has set a benchmark that no patient should have to experience pain estimated to NRS ≥ 4. Equally frustrating is the M-group NRS values at admission to hospital. Since the maximum dose morphine sulphate per kg body weight was given (13.5 ± 3.2 mg, according to our protocol) and led to a NRS value at arriving to hospital of 5.4 ± 1.9, this indicates that these patients with bone fractures are delivered to hospital with moderate to severe pain. This cannot be considered acceptable in modern ambulance care. According to the American Pain Society's (APS) Guidelines for the Treatment of Pain, each patient should receive individual optimal doses of pharmacological pain relief [12]. The results of this clinical study, show significant improvement when using ketamine, however with just morphine sulphate as available analgesic, different organisations guidelines have to contain larger maximal doses than used in this study to patients with different extremity fractures.

This study also demonstrates a significant difference in blood pressure between the times of initial treatment to admission to hospital (Table 3). This increase is to be expected and could be a positive effect in the trauma context if the patient is suspected to be systemic hypovolume. The study also indicates that our patients were Alert or respond to Voice using the AVPU-scale and no patients experienced hallucinations. These findings indicate that staffs who are not anaesthesia trained, does not need to fear that the patients will become unconscious. However, the treatment gave patients in the MK-group a tendency of more nausea and 3 patients vomited. These findings are not consistent with other studies on ketamine and the authors have no explanation for these findings [10,11].

There are some limitations in the present study. First, if the study had been blinded it could have increased the strength of the results. Second, some findings may be due to the given doses and time intervals. However, we believe that the doses are adequate due to previous experiences from emergency care in patients with bone fractures. Third, the sample size might be questionable. Not according to the described power analysis but the limited number of patients might not be sufficient in arguing about unknown safety issues combining the two drugs. Finally, this study does not evaluate whether adding an antiemetic can mitigate the side effects of nausea and vomiting. This question together with the limitations mentioned above should stimulate further studies in this field.

## Conclusion

We conclude that morphine sulphate with the addition of small doses of ketamine provide safe adequate pain relief in patients with bone fractures, with an increase in systolic blood pressure, but without significant side effects.

## Competing interests

The authors declare that they have no competing interests.

## Authors' contributions

PJ made substantial contribution to conception and design to the study. PK participated in data analysis and interpretation. AJ made statistical analysis and substantial contribution to conception and design to the study. All authors read and approved the final manuscript.
